# Structural identification of vasodilator binding sites on the SUR2 subunit

**DOI:** 10.1038/s41467-022-30428-y

**Published:** 2022-05-13

**Authors:** Dian Ding, Jing-Xiang Wu, Xinli Duan, Songling Ma, Lipeng Lai, Lei Chen

**Affiliations:** 1 State Key Laboratory of Membrane Biology, College of Future Technology, Institute of Molecular Medicine, Peking University, Beijing Key Laboratory of Cardiometabolic Molecular Medicine, 100871 Beijing, China; 2 Peking-Tsinghua Center for Life Sciences, Peking University, 100871 Beijing, China; 3 Academy for Advanced Interdisciplinary Studies, Peking University, 100871 Beijing, China; 4 National Biomedical Imaging Center, Peking University, 100871 Beijing, China; 5 Beijing Jingtai Technology Co., Ltd., Beijing, China

**Keywords:** Cryoelectron microscopy, Permeation and transport, Hypertension, Potassium channels, Membrane proteins

## Abstract

ATP-sensitive potassium channels (K_ATP_), composed of Kir6 and SUR subunits, convert the metabolic status of the cell into electrical signals. Pharmacological activation of SUR2- containing K_ATP_ channels by class of small molecule drugs known as K_ATP_ openers leads to hyperpolarization of excitable cells and to vasodilation. Thus, K_ATP_ openers could be used to treat cardiovascular diseases. However, where these vasodilators bind to K_ATP_ and how they activate the channel remains elusive. Here, we present cryo-EM structures of SUR2A and SUR2B subunits in complex with Mg-nucleotides and P1075 or levcromakalim, two chemically distinct K_ATP_ openers that are specific to SUR2. Both P1075 and levcromakalim bind to a common site in the transmembrane domain (TMD) of the SUR2 subunit, which is between TMD1 and TMD2 and is embraced by TM10, TM11, TM12, TM14, and TM17. These K_ATP_ openers synergize with Mg-nucleotides to stabilize SUR2 in the NBD-dimerized occluded state to activate the channel.

## Abstract (web summary)

SUR2-containing KATP channels are drug targets for certain vasodilators. Here, the authors determine high-resolution cryo-EM structures of SUR2 in complex with two vasodilators, P1075 and levcromakalim, uncovering the mechanisms of these drugs.

## Introduction

Physiologically, ATP-sensitive potassium channels (K_ATP_) are inhibited by intracellular ATP and activated by Mg-ADP^1,2^. They are metabolic sensors that couple cellular energy status to the excitability of the plasma membrane^1^. K_ATP_ channels are broadly distributed in many tissues, including the brain, pancreatic islets, and muscles. These channels participate in many physiological processes and are implicated in several pathological conditions. Genetic mutations of K_ATP_ channels in humans lead to a range of diseases, including neonatal diabetes mellitus^3^, hyperinsulinaemic hypoglycemia of infancy^3^, dilated cardiomyopathy^4^, Cantu syndrome^5,6^, familial atrial fibrillation^7^ and intellectual disability myopathy syndrome^8^. K_ATP_ are validated drug targets and are modulated by both pharmaceutical inhibitors and activators, many of which have been approved for use in the clinic.

Functional K_ATP_ channels are hetero-octamers composed of four Kir6 subunits and four SUR subunits. Kir6 are the pore-forming inward rectifier potassium channel subunits encoded by either *KCNJ8* (Kir6.1) or *KCNJ11* (Kir6.2). SURs are type IV ABC transporter-like regulatory subunits encoded by *ABCC8* (SUR1) or *ABCC9* (SUR2)^9^. Additionally, SUR2 has two splicing isoforms, namely SUR2A and SUR2B, which differ in their C-terminal 42 residues. Previous functional studies have established that Kir6 subunits harbor the inhibitory ATP-binding sites, while SUR subunits mediate Mg-ADP activation. SUR subunits are not only targeted by inhibitors, such as insulin secretagogues, but also by activators, such as K_ATP_ openers^10^. Therefore, SUR subunits primarily govern the pharmacological profile of K_ATP_ channels. Moreover, K_ATP_ channels composed of different subunit isoforms have tissue-specific distributions. K_ATP_ channels in the pancreas are mainly formed by the Kir6.2-SUR1 combination, while K_ATP_ channels composed of SUR2 are broadly distributed in muscles, such as Kir6.2-SUR2A combination in the cardiac muscle and Kir6.1-SUR2B combination in the smooth muscles^10^. SUR1, SUR2A, and SUR2B respond differentially to Mg-ADP activation, K_ATP_ openers, and insulin secretagogues^11–15^. Different drug sensitivities of K_ATP_ channel isoforms are exploited to selectively target tissue-specific K_ATP_ subtypes for treating certain diseases.

K_ATP_ openers, a subclass of potassium channel openers (KCO), are small molecules that can activate K_ATP_ in the presence of Mg-ATP or Mg-ADP in patch clamp recordings^16^. The opening of the K_ATP_ channel hyperpolarizes the plasma membrane, reduces the excitability of the cell, and inhibits intracellular calcium signaling. According to their selectivity, K_ATP_ openers are clustered into the following three groups: SUR1-specific openers, such as NN414 (tifenazoxide); SUR2-specific openers, such as P1075 and levcromakalim (Lev); and nonselective openers, such as diazoxide (Supplementary Fig. 1a–h). The activation of the pancreatic K_ATP_ channel by diazoxide is used to block insulin secretion and elevate blood glucose levels for treating hypoglycemia^16^. The SUR2-selective K_ATP_ openers, named “vasodilators”, can relax the smooth muscle of blood vessels, and are clinically used to treat cardiovascular diseases, such as hypertension, angina pectoris, and arrhythmia^16^. Moreover, SUR2-selective K_ATP_ openers also show promise in several clinical applications, including myoprotection, bronchodilation, bladder relaxation, anti-epilepsy^16^, and glaucoma therapeutics^17^.

Recent progress on the determination of K_ATP_ channel structures provides valuable insights into their architecture and regulation^18–26^. However, how SUR2-selective K_ATP_ openers bind and activate the K_ATP_ channel remains enigmatic. To answer these outstanding questions, we embarked on structural studies on SUR2 in complex with its specific K_ATP_ openers.

In this work, we present cryo-EM structures of SUR2A and SUR2B in complex with Mg-nucleotides and P1075 or Levcromakalim in the inward-facing conformation. Our structures reveal the mechanism of SUR2-activator type vasodilators.

## Results

### Structures of SUR2A and SUR2B in the NBD-dimerized state

**Fig. 1:**
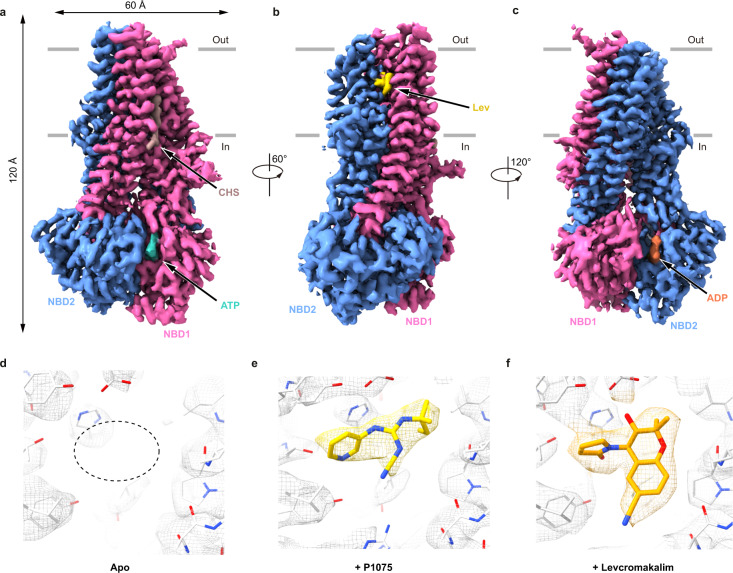
Structure of the SUR2 subunit in complex with K_ATP_ opener and Mg-nucleotides. **a**–**c** Cryo-EM density map of SUR2B in complex with Mg-nucleotides and levcromakalim (Lev), viewed from the side. The approximate position of the lipid bilayer is indicated by gray bars. TMD1-NBD1, TMD2-NBD2, Lev, Mg-ATP, Mg-ADP, and cholesteryl hydrogen succinate (CHS) are colored in pink, blue, yellow, cyan, orange, and brown, respectively. For better visualization of the position of Lev, a fragment of TMD2 in front of Lev was omitted in (**b**); the residual detergent densities are also omitted. **d** Empty K_ATP_ opener-binding site in the cryo-EM map of the SUR2B subunit in complex with Mg-nucleotides is outlined by dashed lines. The map is shown as mesh and the protein is shown as sticks. **e** P1075 density (yellow) in the SUR2B subunit (gray). **f** Lev density (orange) in the SUR2B subunit (gray).

Rat SUR2 subunits share 96.7% sequence identity with their human counterparts. They can be activated by K_ATP_ openers such as P1075 and Lev as well (Supplementary Fig. 1). Therefore, we used them to study the mechanism of SUR2-specific K_ATP_ openers. We initially focused on the structures of SUR2-containing hetero-octameric K_ATP_ channels in the Mg-nucleotides-activated state. However, despite extensive trials, we could not obtain the high-resolution cryo-EM structures, probably due to the instability of the complex and high degree of conformational heterogeneity at these conditions. Because previous studies have established that the SUR subunit alone harbors the binding sites of K_ATP_ openers^14^, we then used isolated rat SUR2A and SUR2B subunits for structural studies of the mechanism of K_ATP_ openers (Supplementary Fig. 1). It is reported that these drugs bind to SUR2 with slowed off-rates in the presence of Mg-ADP or Mg-ATP^27^, therefore, we also supplemented cryo-EM samples with Mg-nucleotides to enhance the affinity of drugs. We first solved the cryo-EM structure of SUR2B in the presence of Mg-ADP/Mg-ATP but without K_ATP_ openers to the resolution of 3.4 Å (Supplementary Fig. 2). The ABC transporter core of SUR2 shows an occluded state, which is similar to SUR1 in Mg-nucleotide-bound K_ATP_ structures^18,22,25,26^. However, the density of TMD0 was missing due to its flexibility, suggesting that TMD0 was unstable without co-assembly with the Kir6 channel. To understand how K_ATP_ openers are engaged with SUR2, we further solved the structure of SUR2B in complex with P1075, SUR2B in complex with Lev, and SUR2A in complex with P1075 to the resolutions of 3.3 Å, 3.1 Å, and 3.3 Å, respectively (Supplementary Figs. 3, 4, 5 and Supplementary Table 1). These four high-resolution maps allowed us to unambiguously identify the drug binding sites in the transmembrane domain (Fig. 1). These structures are all in the “occluded” conformation and highly similar with each other (Supplementary Fig. 6a, b), except that different ligands were bound, suggesting SUR2 binds P1075 and Lev via a lock-and-key mechanism. These occluded structures show large conformational changes compared with the inward-facing structure with glibenclamide (GBM) bound^24^ (Supplementary Fig. 6c, d).

### P1075-binding site on SUR2

**Fig. 2:**
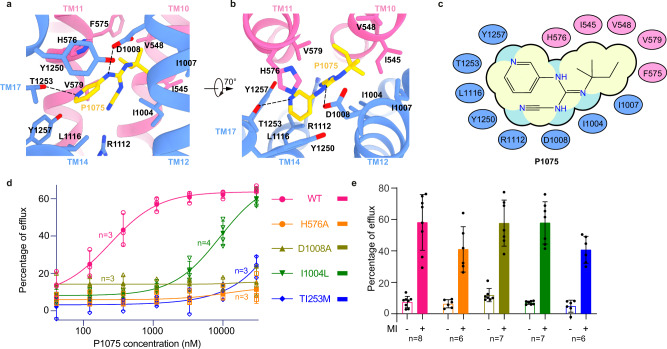
P1075-binding site in SUR2B. **a**, **b** Close-up views of the P1075-binding site. TMD1 and TMD2 are colored in pink and blue, respectively. P1075 (yellow) and residues that interact with P1075 are shown as sticks. Putative H-bonds are shown as dashed lines. **c** Cartoon representation of the interaction between P1075 and SUR2B. The key residues on TMD1 and TMD2 are shown as pink and blue ovals, respectively. **d** The dose–response activation curves of SUR2B-Kir6.2 K_ATP_ channel by P1075 measured by Rb+ efflux assay. Curves were fitted to the Hill equation. Data are presented as mean values ± SD The numbers of independent experiments of WT, H576A, D1008A, I1004L, and T1253M are 3, 3, 3, 4, and 3, respectively. **e** Effects of metabolism inhibitors (MI, 1 mM 2-deoxy-D-glucose, and 3 μM oligomycin) on KATP constructs containing various SUR2B mutants. Data are presented as mean values ± SD. The numbers of independent experiments of WT, H576A, D1008A, I1004L, and T1253M are 8, 6, 7, 7, and 6, respectively. Source data are provided as a Source Data file.

P1075 (N-Cyano-N’-(1,1-Dimethylpropyl)-N”-(3-Pyridinyl)Guanidine) is a cyanoguanidine type K_ATP_ opener that has a similar structure to pinacidil, an FDA approved anti-hypertensive drug (Supplementary Fig. 1a, b). P1075 robustly activates both SUR2A and SUR2B^28^. The cryo-EM maps of P1075 in complex with both SUR2A and SUR2B show that P1075 sits in the middle of SUR2 TMD and is embraced by TM10, TM11, TM12, TM14, and TM17 helices (Fig. 2a, b). We named this pocket the “K_ATP_ channel opener-binding site” (KCOS). The binding energy of P1075 to SUR2B was estimated to be −44.22 kcal/mol using computational methods. The binding is stable during 100 ns MD simulation (Supplementary Fig. 6e). In detail, the cyano group of P1075 interacts with R1112 on the TM14 of SUR2B; its pyridine group interacts with Y1257, T1253, and Y1250 on TM17, and L1116 on TM14; its middle guanidine group interacts with H576 on TM11 and D1008 on TM12; and its dimethylpropyl group inserts into a hydrophobic pocket surrounded by I545 and V548 on TM10, F575 and V579 on TM11, and I1004 and I1007 on TM12 (Fig. 2c). We used site-directed mutagenesis and the Rb^+^ efflux assay to interrogate the functions of these residues on P1075-induced channel activation (Fig. 2d, e). All the mutants showed obvious Mg-ADP activation, (Fig. 2e), suggesting that these SUR2B mutants are well folded, although the activation of H576A or T1253M is slightly reduced (Fig. 2e). We found the mutations on two conserved residues, H576A or D1008A, almost completely abolished the activation by P1075, indicating the interactions between P1075 and H576 or D1008 are essential for P1075 activation (Fig. 2d). Because P1075 is a SUR2-selective K_ATP_ opener, we searched for residues that are involved in P1075 binding but are different between SUR1 and SUR2. I1004 and T1253 in SUR2 are Leu and Met in SUR1, respectively. Switching I1004 to Leu or T1253 to Met might introduce steric hindrance to KCOS and thus reduce the binding of P1075 (Supplementary Fig. 6g). Indeed, we found that mutation of I1004L or T1253M in SUR2 reduced the potency of P1075, with I1004L having a more profound effect (Fig. 2d), suggesting that these residues contribute to the subtype selectivity of P1075. This is also in agreement with previous findings showing that the reverse mutations of SUR1 (T1285L and M1289T) enhance the affinities of pinacidil, P1075, and thioformamide aprikalim (Supplementary Fig. 1c) towards SUR1^29^.

### Lev-binding site on SUR2B

**Fig. 3:**
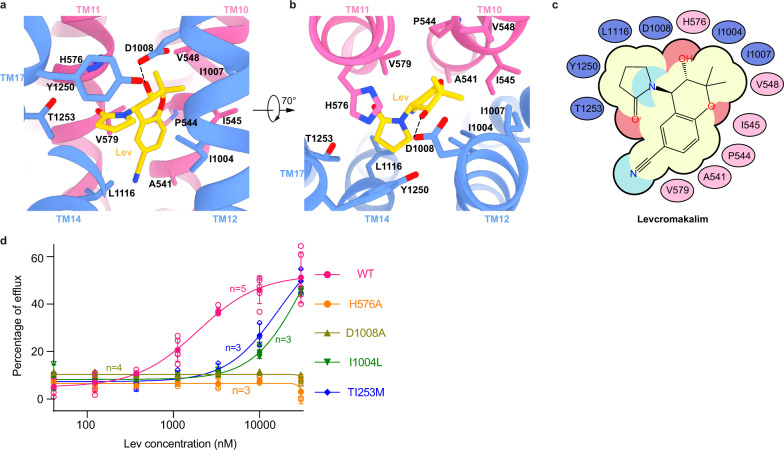
Lev-binding site in SUR2B. **a**, **b** Close-up views of the Lev-binding site. TMD1 and TMD2 are colored in pink and blue, respectively. Lev (yellow) and residues that interact with Lev are shown as sticks. Putative H-bonds are shown as dashed lines. **c** Cartoon representation of the interactions between Lev and SUR2B. The residues on TMD1 and TMD2 are represented as pink and blue ovals, respectively. **d** Dose–response activation curves of SUR2B- Kir6.2 K_ATP_ channel by Lev measured by Rb^+^ efflux assay. Curves were fitted to the Hill equation. Data are presented as mean values ± SD. The numbers of independent experiments of WT, H576A, D1008A, I1004L and T1253M are 5, 3, 4, 3 and 3, respectively. Source data are provided as a Source Data file.

Lev ((3 S,4 R)-3-hydroxy-2,2-dimethyl-4-(2-oxopyrrolidin-1-yl)-3,4-dihydrochromene-6-carbonitrile) (Supplementary Fig. 1e) is the bioactive isomer of cromakalim (Supplementary Fig. 1f), a SUR2-selective K_ATP_ opener. Lev has a dihydrochromene core and an oxopyrrolidin branch. We found that Lev also binds in the KCOS of SUR2. The binding free energy of Lev to SUR2B was estimated to be −42.98 kcal/mol using computational methods. The binding is stable during 100 ns MD simulation (Supplementary Fig. 6f). Specifically, the carbonyl group on the oxopyrrolidin group of Lev interacts with T1253 on TM17; its pyrrolidine ring hydrophobically interacts with L1116 on TM14, Y1250 on TM 17, and I1004 on TM12; the dihydrochromene group hydrophobically interacts with P544, I545, A541, and V548 on TM10, I1007 and I1004 on TM12, and V579 on TM11; its hydroxyl group interacts with D1008 on TM12 and H576 on TM11 (Fig. 3a–c). We found that D1008A and H576A mutations largely diminished the activation of Lev (Fig. 3d), while I1004L and T1253M mutations reduced the potency of Lev to a similar extent (Fig. 3d and Supplementary Fig. 6h). This agrees with previous findings showing that T1285L and M1289T mutations in SUR1 increased the amount of insulin secretagogue glibenclamide displaced by levcromakalim and rilmakalim (Supplementary Fig. 1g)^29^.

### Nucleotide-binding sites of SUR2 in the Mg-ADP/Mg-ATP bound state

**Fig. 4:**
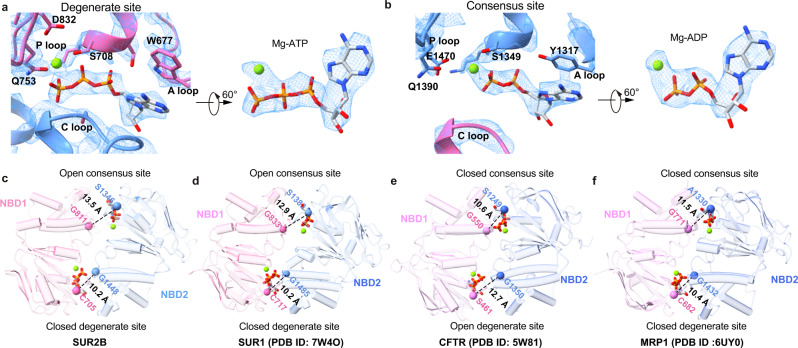
Structural comparisons of the NBDs of ABCC family members. **a**, **b** Close-up views of EM densities at the degenerate site and the consensus sites. **c** Bottom view of the NBD1 and NBD2 of SUR2B in the Mg-nucleotides and P1075-bound state. ATP and ADP molecules are shown as sticks. Mg^2+^ are shown as green spheres. The color scheme is the same as that in Fig. 1. The Cα distances between glycine in the Walker A motif and serine (cysteine at the degenerate site) in the ABC signature motif are shown as dashed lines. **d** NBD structures of SUR1. View of SUR1 (PDB ID: 7W4O) NBDs from the bottom. ATP and ADP molecules are shown as sticks. Cα distances between glycine in the Walker A motif and serine (cysteine at the degenerate site and alanine at the consensus site) in the ABC signature motif are shown as dashed lines. **e** NBD structures of CFTR. View of CFTR (PDB ID: 5W81) NBDs from the bottom. ATP and ADP molecules are shown as sticks. Cα distances between glycine in the Walker A motif and serine in the ABC signature motif are shown as dashed lines. **f** NBD structures of MRP1. View of MRP1 (PDB ID: 6UY0) NBDs from the bottom. ATP and ADP molecules are shown as sticks. Cα distances between glycine in the Walker A motif and serine (cysteine at the degenerate site and alanine at the consensus site) in the ABC signature motif are shown as dashed lines.

We observed that Mg-nucleotides are bound to both nucleotide-binding sites of SUR2 (Fig. 4a, b). SUR subunits have one consensus nucleotide-binding site and one degenerate site^2^. Although we prepared the SUR2B + P1075 samples using Mg-ATP alone, while other SUR2 samples using both Mg-ADP and Mg-ATP, we found that Mg-ATP was bound in the degenerate site and Mg-ADP was bound in the consensus site in all of the structures. It is possible that in the SUR2B + P1075 sample, the ADP might come from the contamination of the Mg-ATP stock. This was also observed previously in SUR1-containing K_ATP_ channel^18^. In the degenerate site, the Mg-coordinated ATP interacts extensively with both NBD1 and NBD2 (Fig. 4a). The adenosine group of ATP stacks with W677 on the A-loop, and the triphosphate of ATP is embraced by the P-loop of NBD1 (Fig. 4a). Moreover, Q753 on the Q-loop, D832 on the Walker B motif, S708 on the P-loop, and β, γ-phosphates coordinate the Mg^2+^ ion (Fig. 4a). In addition, the C-loop of NBD2 is in close proximity to ATP, and the positive dipole of helix 5 on NBD2 points to the triphosphate group of ATP (Fig. 4a). As a result, ATP is tightly sandwiched between NBD1 and NBD2, and the degenerate site is fully closed (Fig. 4a). In the consensus site, the Mg-coordinated ADP molecule is bound to the NBD2, interacting with Y1317 on the A-loop, and multiple residues on the P-loop. The Mg^2+^ ion is coordinated by the β phosphate of ADP, Q1390 on the Q loop, and E1470 on the Walker B motif of the NBD2 (Fig. 4b). However, as the C-loop of the opposing NBD1 is away from Mg-ADP (Fig. 4b), the consensus site is still open. The asymmetric dimerization of SUR2 NBDs observed here is akin to the SUR1 in the Mg-ADP bound state^22^(Fig. 4c, d) or the Mg-ATP/Mg-ADP bound state^18,25,26^ but is different from structures of other ABCC family members with dimerized NBDs, such as E1372Q mutant of CFTR (PDB ID: 5W81)^30^, which has a closed consensus site but an open degenerate site (Fig. 4e) or MRP1 under active turnover condition (PDB ID: 6UY0)^31^, which has both consensus site and degenerate site closed (Fig. 4f). The asymmetric dimerized NBD arrangement of SUR2 is also different from the heterodimeric ABC transporter TM287/288, of which both NBDs are either not engaged in the AMP-PNP-bound state (PDB ID: 4Q4A)^32^ or fully closed in the ATP-bound state (PDB ID: 6QV0)^33^.

The C-terminal 42 residues of SUR2 fold into α7 to α9, which is part of NBD2 (Supplementary Fig. 7 and 8). Although these residues are different between SUR2A and SUR2B, we found that their structures are highly similar in the Mg-ADP/Mg-ATP and P1075-bound states with an RMSD of 0.327 Å (Supplementary Fig. 8a, b).

## Discussions

The KCOS identified here is surrounded by TM10, TM11, TM12, TM14, and TM17, which is consistent with previous data showing that T1059–L1087 (TM13) and R1218–N1320 (TM17) are important for the binding of K_ATP_ openers^34^. Both P1075 and levcromakalim bind in the KCOS of SUR2, which is in agreement with the competitive binding behavior between P1075 and nicorandil or diazoxide on SUR2B^35^. Because diazoxide can activate both SUR2B and SUR1, diazoxide probably binds to SUR2B and SUR1 at the same site. Indeed, our accompanying work on SUR1 showed that the diazoxide analogue NN414 binds at a similar position in SUR1^26^ (Supplementary Fig. 8c, d). These structures collectively suggest that the KCOS observed here is a hotspot for the binding and action of K_ATP_ openers. When the structure of SUR2-P1075 is aligned to the structure of SUR1-NN414^26^, we found that the overall conformations of the ABC transporter module are similar but the KCOS in SUR1 with NN414 bound is more expended than SUR2. Particularly, the Cα distance between H576 and D1008 in SUR2 is 10.2 Å while the Cα distance between H584 and D1031 in SUR1 is 12.3 Å (Supplementary Fig. 8d). These structural observations collectively suggest that not only the types of residues surrounding the KCOS but also the conformations of TM helices forming the KCOS contribute to the subtype-selectivity of K_ATP_ openers.

Moreover, our structures also shed light on the structure-activity relationship of SUR2-specific K_ATP_ openers. P1075 is a high-affinity analogue of pinacidil. The conversion of trimethylpropyl group of pinacidil into dimethylpropyl group of P1075 (Supplementary Fig. 1a) results in a more favorable hydrophobic packing between these groups and the hydrophobic pocket on SUR2, formed by I545, V548, V579, and I1004 (Fig. 2), leading to the marked increase of potency^13^. Our mutagenesis experiments showed that I1004 and T1253 of SUR2 are important for the potencies of both P1075 and levcromakalim because mutations of them into the corresponding residues of SUR1 (I1004L and T1253M) decrease drug potencies.

Because NBD1 of SUR has a high affinity for ATP^22,36^, it is probably constantly occupied by Mg-ATP inside the cell. Upon binding of Mg-ADP to NBD2 of SUR2, NBD1 and NBD2 dimerize asymmetrically, with the degenerate site fully closed while the consensus site remains open. The dimerization of NBDs further drives the closure of TMD1 and TMD2 and the occlusion of TMD. K_ATP_ openers interact with both TMD1 and TMD2 and thus also promote the TMD into the occluded state, suggesting the positive cooperativity between the Mg-nucleotide and the K_ATP_ opener on the activation of K_ATP_^14,28^. In contrast, insulin secretagogues bind to a site embraced by TM7, TM8, TM11, TM16, and TM17 in SUR1^23^ and SUR2^24^, which is ~20 Å away from KCOS (Supplementary Fig. 6d). Insulin secretagogues stabilize the SUR in the inward-facing conformation (Supplementary Fig. 6c) and thus allosterically inhibit the binding of the K_ATP_ opener^35^ and the conformational changes induced by Mg-ADP. Although the Mg-nucleotide-dependent conformational switch of SURs from inward-facing to occluded state resembles other ABC exporters^37,38^, SURs are not found to be able to populate the outward-facing conformation yet. Moreover, contrasting to other ABCC family members that rely on the binding of Mg-ATP to consensus site for their activity, exemplified by CFTR^39,40^, the NBD-dimerization of SUR is largely induced by Mg-ADP binding to the consensus site of NBD2^2^, emphasizing that SURs are dedicated sensor for Mg-ADP.

**Fig. 5:**
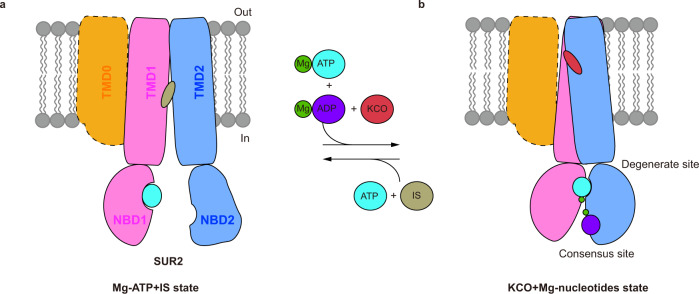
Model for SUR2-containing K_ATP_ channel activation by Mg-nucleotides and K_ATP_ opener. **a**, **b** Side view of the cartoon model of SUR2 subunits. TMD0, TMD1-NBD1, TMD2-NBD2, Mg^2+^, ATP, ADP, insulin secretagogue (IS), and K_ATP_ opener (KCO) are colored in orange, pink, blue, dark green, cyan, purple, gray and red, respectively. TMD0 is outlined as dashes because it is invisible in cryo-EM maps.

Previous structural studies have established that the NBD-dimerized occluded conformation of SUR1 is associated with K_ATP_ channel opening^19,22,25,26^ and the NBD-separated inward-facing conformation of SUR1 is associated with K_ATP_ inhibition^20–22,41^. The current work further extends these structural observations onto SUR2, suggesting a common activation mechanism of both SUR1-containing and SUR2-containing K_ATP_ channels (Fig. 5). Although SUR2A and SUR2B differ in Mg-ADP activation and K_ATP_ opener sensitivity^11–15^, their structures are highly similar in the Mg-nucleotides and K_ATP_ opener-bound state (Supplementary Fig. 8a, b). Therefore, we speculate the structures of SUR2A and SUR2B might have structural variations in the NBD-separated inward-facing conformation and such structural difference leads to the functional differences between SUR2A and SUR2B.

Taken together, the structures presented here provide a framework to further understand the mechanism of vasodilators that targets SUR2-containing K_ATP_ channels and pave the way for the rational design and optimization of new K_ATP_ openers for the treatment of related diseases.

## Methods

### Cell lines

FreeStyle 293 F (Thermo Fisher Scientific) suspension cells were cultured in SMM 293-TI (Sino Biological Inc.) supplemented with 1% FBS at 37 °C, with 6% CO_2_ and 70% humidity. Sf9 insect cells (Thermo Fisher Scientific) were cultured in Sf-900 III SFM medium (Thermo Fisher Scientific) at 27 °C. AD293 cells (Agilent) were cultured in DMEM basic (Thermo Fisher Scientific) supplemented with 10% fetal bovine serum (FBS) at 37 °C, with 6% CO_2_ and 70% humidity.

### Expression constructs

cDNAs of SUR2A and SUR2B from *Rattus norvegicus* were cloned into C-terminal GFP tagged BacMam expression vector which also contains two Strep tags and one His8 tag. For functional studies, Kir6.2 was cloned into a modified C-terminal GFP-tagged BacMam expression vector and SUR2 mutants were cloned into a BacMam expression vector without tags as described previously^22^.

### Electrophysiology

K_ATP_ constructs were transfected into FreeStyle 293-F cells using polyethylenimine at a cell density of 1 × 10^6^ cells/ml. Cells were cultured in FreeStyle 293 Expression Medium with 1% FBS for 24–36 h before recording. Macroscopic currents were recorded using inside-out mode at +60 mV through and an Axon-patch 200B amplifier (Axon Instruments, USA). Patch electrodes were pulled by a horizontal micro-electrode puller (P-1000, Sutter Instrument Co, USA) to tip resistance of 1.0–3.0 MΩ. Pipette solution containing (mM): 140 KCl, 1.2 MgCl_2_, 2.6 CaCl_2_, 10 HEPES (pH 7.4, NaOH) and bath solution containing (mM): 140 KCl, 10 EGTA, 1 MgCl_2_, 10 HEPES (pH 7.4, NaOH) were used for measuring inhibitory effect of Mg-ATP. For measuring the inhibitory effects of ATP without Mg^2+^, bath solution containing (mM): 140 KCl, 10 EDTA, and 10 HEPES (pH 7.4, NaOH) was used. Signals were acquired at 5 kHz and low-pass filtered at 1 kHz. Data were further analyzed with pClampfit 10.0 software.

### Rb^+^ efflux assay

K_ATP_ constructs were transfected into AD293 cells using polyethylenimine. Cells were cultured in DMEM medium with 10% FBS for 24 h before being transferred into a 96-well plate. Cells were incubated overnight in a medium containing 6 mM RbCl. The next day, cells were washed in Ringer’s solution (118 mM NaCl, 10 mM HEPES (pH 7.4), 25 mM NaHCO_3_, 4.7 mM KCl, 1.2 mM KH_2_PO_4_, 2.5 mM CaCl_2_, and 1.2 mM MgSO_4_) and incubated with Ringer’s solution with KCOs at different concentrations for 20 min. To measure the effect of metabolism inhibitors (MI), cells were pre-incubated with MI and 6 mM RbCl for 20 min, and then incubated with Ringer’s buffer with MI for 20 min after washing out excessive RbCl. Efflux solution was collected, and the cells were lysed in Ringer’s solution containing 1% Triton X-100. Rb^+^ in the efflux solution and cell lysate was counted using Ion Channel Reader 8000 (Aurora Group Company).

### Expression and purification of SUR2 subunits

SUR2 subunits were expressed using the BacMam system as described previously with minor modifications^19^. Briefly, cells were harvested 48 h post-infection and membrane pellets were purified as described previously^22^. For purification, membrane pellets were homogenized in TBS (20 mM Tris and 200 mM NaCl) and then solubilized in 1% GDN and 0.05% CHS for 30 min at 4 °C. Unsolubilized material was removed by centrifugation at 100,000 g for 30 min. The supernatant was supplemented with 1 mM ATP and 1 mM MgCl_2_ and loaded onto Streptactin Beads 4FF (Smart Lifesciences). The beads were washed with buffer A (TBS with 50 µM GDN and 1 mM ATP) plus 1 mM MgCl_2_ and protein was eluted with buffer A plus 10 mM desthiobiotin. GFP tags were removed by PreScission protease. To purify the SUR2B protein for Mg-nucleotides + Lev state and Mg-nucleotide-bound state, proteins were supplemented with 2 mM ADP + 2 mM MgCl_2_ and concentrated by 100-kDa cut-off concentrator (Sartorius) and loaded onto Superose 6 increase (GE Healthcare) running in TBS with 50 μM GDN, 2 mM ADP and 2 mM MgCl_2_. To purify the protein of SUR2B in the Mg-nucleotides + P1075 state, protein was loaded onto Superose 6 increase running in buffer A with 1 mM MgCl_2_.

### Cryo-EM sample preparation

Purified SUR2B proteins were further supplemented with 3 mM fluorinated Fos-Choline-8 (FFC), 8 mM MgCl_2_, 8 mM ATP (Sigma) and 200 μM P1075 (Tocris Bioscience) for Mg-nucleotides + P1075 state; 8 mM MgCl_2_ and 8 mM ADP (Sigma) for Mg-nucleotides state; 8 mM MgCl_2_, 8 mM ADP and 200 μM Lev (MedChemExpress) for Mg-nucleotides + Lev state. Purified SUR2A proteins were supplemented with 3 mM FFC, 8 mM MgCl_2_, 8 mM ADP and 200 μM P1075 for Mg-nucleotides + P1075 state. Cryo-EM sample was loaded on to glow-discharged Quantifoil 0.6/1 gold grids and frozen as described previously^19^.

### Cryo-EM data acquisition

Cryo-grids were screened on Talos Arctica microscope (Thermo Fisher Scientific) operated at 200 kV and grids in good quality were transferred into Titan Krios microscope (Thermo Fisher Scientific) operated at 300 kV for data acquisition. Images were collected using K2 camera (Gatan) mounted post a Quantum energy filter with 20 eV slit and operated under super-resolution mode with a pixel size of 0.821 Å at the object plane. Defocus values were set to range from −1.5 μm to −2.0 μm for data collection. Data were acquired by Serial-EM-3.6.11. The dose rate on the detector was 11.9 e^−^s^−1^A^−2^. And the total exposure was 51.6 e^−^A^−2^. Each 4.35 s movie was dose-fractioned into 30 frames.

### Image processing

Collected movies were gain-corrected, motion-corrected, exposure-filtered, mag-distortion-corrected and binned with MotionCor2-1.3.2^42^, producing dose-weighted, and summed micrographs with pixel size 0.821 Å. CTF models of dose-weighted micrographs were determined using GCTF-1.18^43^. Auto-picking was done by Gautomatch-0.56 (developed by Kai Zhang, MRC-LMB). Auto-picked particles were extracted from dose-weighted micrographs by a binning factor of 2. SUR2B datasets of Mg-nucleotide + P1075 state and Mg-nucleotide state were subjected to 2D classification using RELION 3.0^44^. Particles yielding from 2D classification (Mg-nucleotide + P1075 state and Mg-nucleotide state of SUR2B) or auto-picking (Mg-nucleotide + P1075 state of SUR2A and Mg-nucleotide + Lev state of SUR2B) were subjected to 50 iterations *K* = 1 global search 3D classification with an angular sampling step of 7.5° to determine the initial alignment parameters using initial model generated by cryoSPARC-3.1.0^45^ as reference. *K* = 4 multi-reference local angular search 3D classification was performed with an angular sampling step of 3.75° and a search range of 15°. The multi-references were generated using the initial model low-pass filtered to 8, 15, 25, and 35 Å, respectively. Particles from selected 3D classes were re-centered and re-extracted from summed micrographs with a binning factor of 1.6 (to yield the pixel size of 1.31 Å). Particles were subjected to another round of initial model generation with *n* = 3 using cryoSPARC-3.1.0^45^. Particles from good model were further refined against the initial model using non-uniform refinement in cryoSPARC-3.1.0 to reach 3.3 Å (Mg-nucleotide + P1075 state of SUR2B), 3.4 Å (Mg-nucleotide state of SUR2B), 3.1 Å (Mg-nucleotide + Lev state of SUR2B), and 3.3 Å (Mg-nucleotide + P1075 state of SUR2A), respectively.

### Model building

Maps were converted to MTZ files by PHENIX-1.18rc1-3777^46^. We used structures of the SUR1 subunit (5YWD and 6JB1) of our previous K_ATP_ structure as the initial model for NBD-dimerized states and inward-facing states, respectively. The homologous SUR2 structures were generated using SWISS-MODEL^47^ and docked into the cryo-EM map with UCSF Chimera-1.14^48^. Models were manually rebuilt in Coot-0.9.2^49^ and further refined by PHENIX-1.18rc1-3777^46^. Figures were prepared with Pymol-1.7.0.5 (Schrodinger, LLC.) and UCSF ChimeraX-0.91^49^.

### Computational methods to optimize ligand binding pose

The molecular docking was performed using LeDock (version Omega) software^50^ to predict the binding modes of compounds. We completed the protein preparation using the LePro module (with the default values) and the ligand preparation using RDKit (version 2021.03.4) (from open-source cheminformatics; https://www.rdkit.org). The docking was run with the default parameter values of the LeDock (version Omega). We selected the best pose for future evaluation and analysis.

To validate which pose is the most reasonable binding conformation in the pocket, we ran MD (Molecular Dynamics) simulations to evaluate the stability of the docking poses. The MD simulations were performed on graphics processor units (GPUs) with AMBER20’s GPU-accelerated PMEMD simulation code^51^. All MD simulations used the AMBER ff14SB Force field^52^ for the protein and gaff2 for ligand. The complex systems were placed in a box of TIP3P water model^53^ and simulated at a constant temperature of 298.15 K. All bonds containing hydrogens were constrained with the SHAKE algorithm. We also calculated the MM/GBSA (Molecular Mechanics/Generalized Born Surface Area) to estimate the binding energy. We simulated 10 ns for MM/GBSA and made the free energy decomposition. Further, we performed a 100 ns MD simulation to evaluate system stability.

### Quantification and statistical analysis

Global resolution estimations of cryo-EM density maps are based on the 0.143 Fourier Shell Correlation criterion^54^. The local resolution map was calculated using cryoSPARC-3.1.0^45^. Rb^+^ efflux assay curves were fitted to the Hill equation using GraphPad Prism 5.0. Electrophysiological data reported were analyzed with pClampfit 10.0 software, calculated with Microsoft Excel and GraphPad Prism 5.0. The number of biological replicates (N) and the relevant statistical parameters for each experiment (such as mean or standard error) are described in figure legends. No statistical methods were used to pre-determine sample sizes.

### Reporting summary

Further information on research design is available in the Nature Research Reporting Summary linked to this article.

## Supplementary information

Supplementary Information

Reporting Summary

## Data Availability

The data that support the findings of this study are available from the corresponding author upon reasonable request. The structure data generated in this study have been deposited in EMDB and PDB as follows: SUR2B in complex with Mg-ATP/ADP: PDB: 7VLR, EMDB-32024; SUR2B in complex with Mg-ATP/ADP and P1075: PDB: 7VLS, EMDB-32025; SUR2B in complex with Mg-ATP/ADP and Lev: PDB: 7VLT, EMDB-32026; SUR2A in complex with Mg-ATP/ADP and P1075: PDB: 7VLU, EMDB-32027. PDB ID: 7W4O, 5W81, 6UY0, 7MIT, 5YWD and 6JB1 are already available on Protein Data Bank. Source data are provided with this paper.

## Source data

Source Data

## Materials availability

Reagents generated in this study will be made available on request, but we may require a payment and/or a completed Materials Transfer Agreement if there is potential for commercial application.
