# Supplementary material for: Structural identification of vasodilator binding sites on the SUR2 subunit
Source: Nat Commun. 2022 May 13;13:2675. doi: 10.1038/s41467-022-30428-y (PMC9106677; doi:10.1038/s41467-022-30428-y)
Supplement: Supplementary file 1 — Supplementary Information [file 41467_2022_30428_MOESM1_ESM.pdf]

Supplementary information for

Structural identification of vasodilator binding sites on the SUR2 subunit

Dian Ding, Jing-Xiang Wu, Xinli Duan, Songling Ma, Lipeng Lai, Lei Chen*

*To whom correspondence should be addressed: Lei Chen (chenlei2016@pku.edu.cn)

This file includes:
Supplementary Figs. 1-8
Supplementary table 1

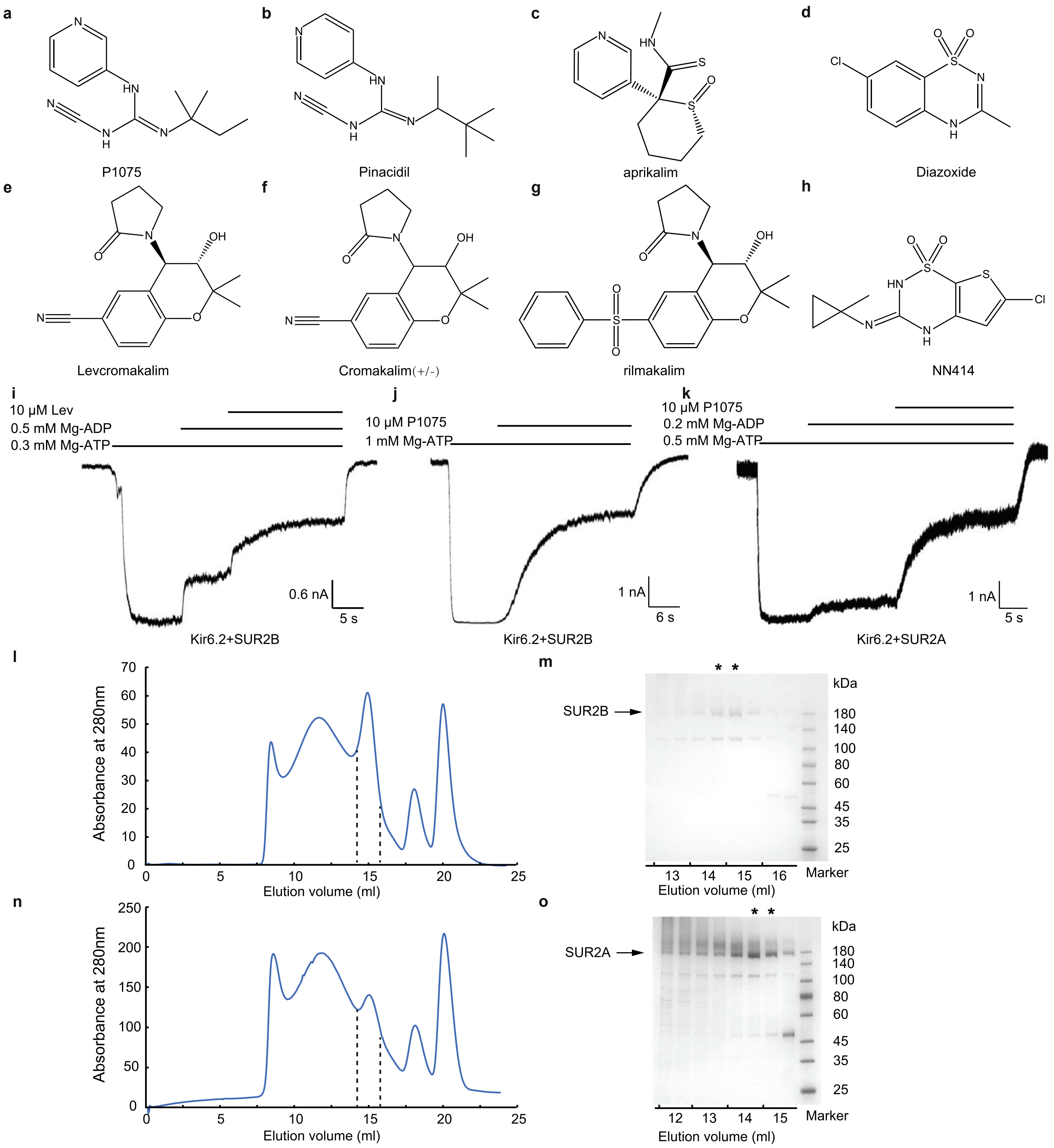

Supplementary Fig. 1: Characterization of K_{ATP} openers and SUR2A and SUR2B. a-h Chemical structure of P1075 (**a**), levcromakalim (Lev) (**b**), aprikalim (**c**), diazoxide (**d**), pinacidil (**e**), cromakalim (**f**), rilmakalim (**g**), and NN414 (**h**). **i** Inhibition of Mg-ATP and activation effect of Mg-ADP and Lev on the SUR2B-Kir6.2 K_{ATP} channel in the inside-out mode. **j** Inhibition of Mg-ATP and activation effect of P1075 on the SUR2B-Kir6.2 K_{ATP} channel. **k** Inhibition of Mg-ATP and activation effect of Mg-ADP and Lev on the SUR2A-Kir6.2 K_{ATP} channel in inside-out mode. **l** Size exclusion chromatography (SEC) elution profile of SUR2B protein. The pooled fractions between the dashed lines were used for cryo-EM sample preparation. **m** SDS-PAGE of purified K_{ATP} channel corresponding to SEC fractions in (**l**). Fractions labeled with asterisks were pooled and concentrated for cryo-EM grids preparation. Experimet was repeated independently more than 3 times with similar results. **n** SEC elution profile of SUR2A protein. The pooled fractions between the dashed lines were used for cryo-EM sample preparation. **o** SDS-PAGE of the purified K_{ATP} channel corresponding to the SEC fractions in (**n**). The fractions labeled with asterisks were pooled and concentrated for cryo-EM grid preparation. Experimet was repeated independently more than 3 times with similar results.

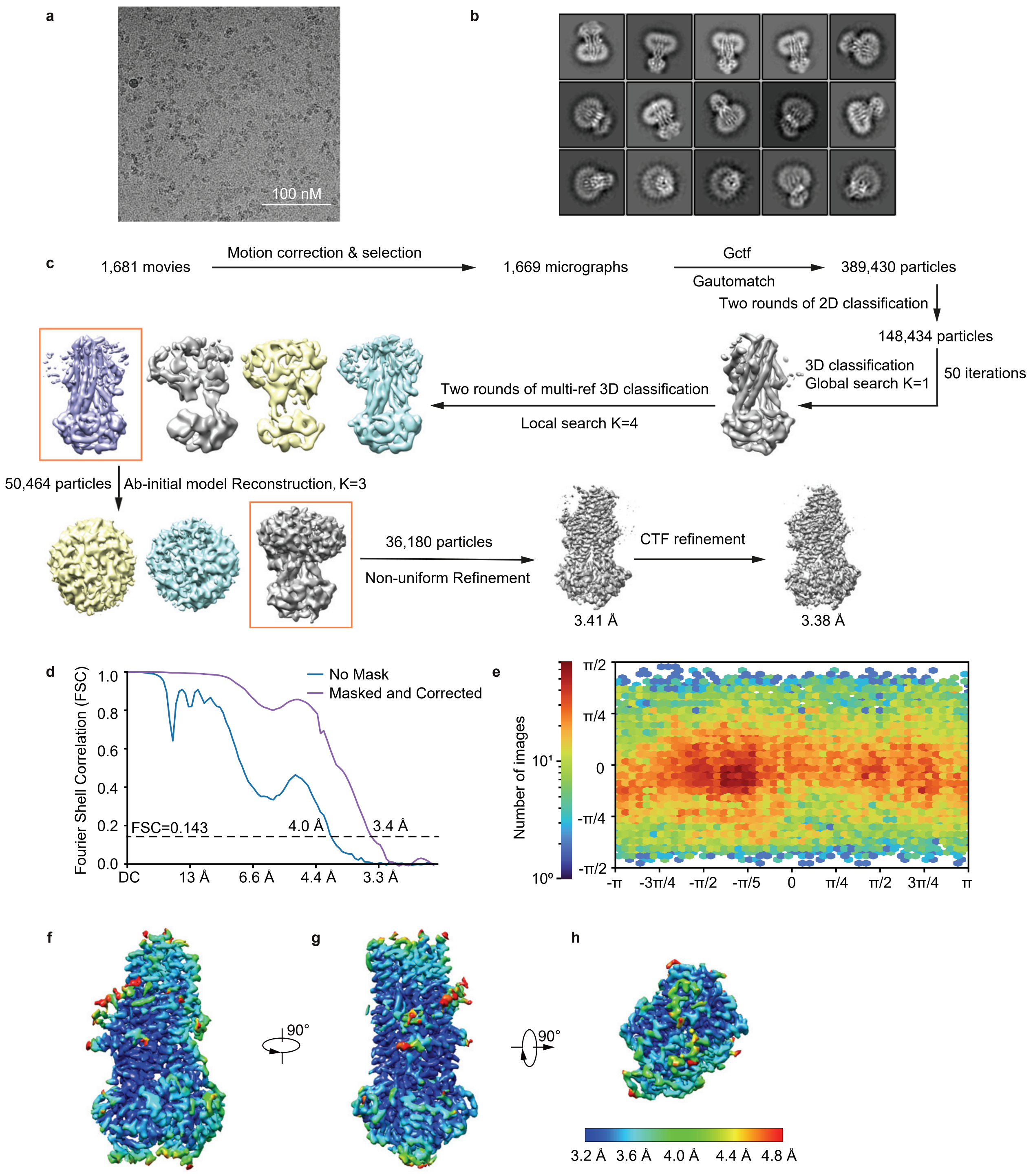

Supplementary Fig. 2: Workflow for cryo-EM data processing of the SUR2B in complex with Mg-nucleotides. a Representative image from a dataset consisting of 1,669 motion corrected micrographs. Experimet was repeated independently more than 3 times with similar results. **b** Representative two-dimensional class averages of SUR2B. **c** EM data processing workflow. **d** Resolution estimation of the SUR2B map, based on the criterion of the FSC 0.143 cut-off. **e** Angular distribution of the final reconstruction. **f-h** Local resolution map of the final density map.

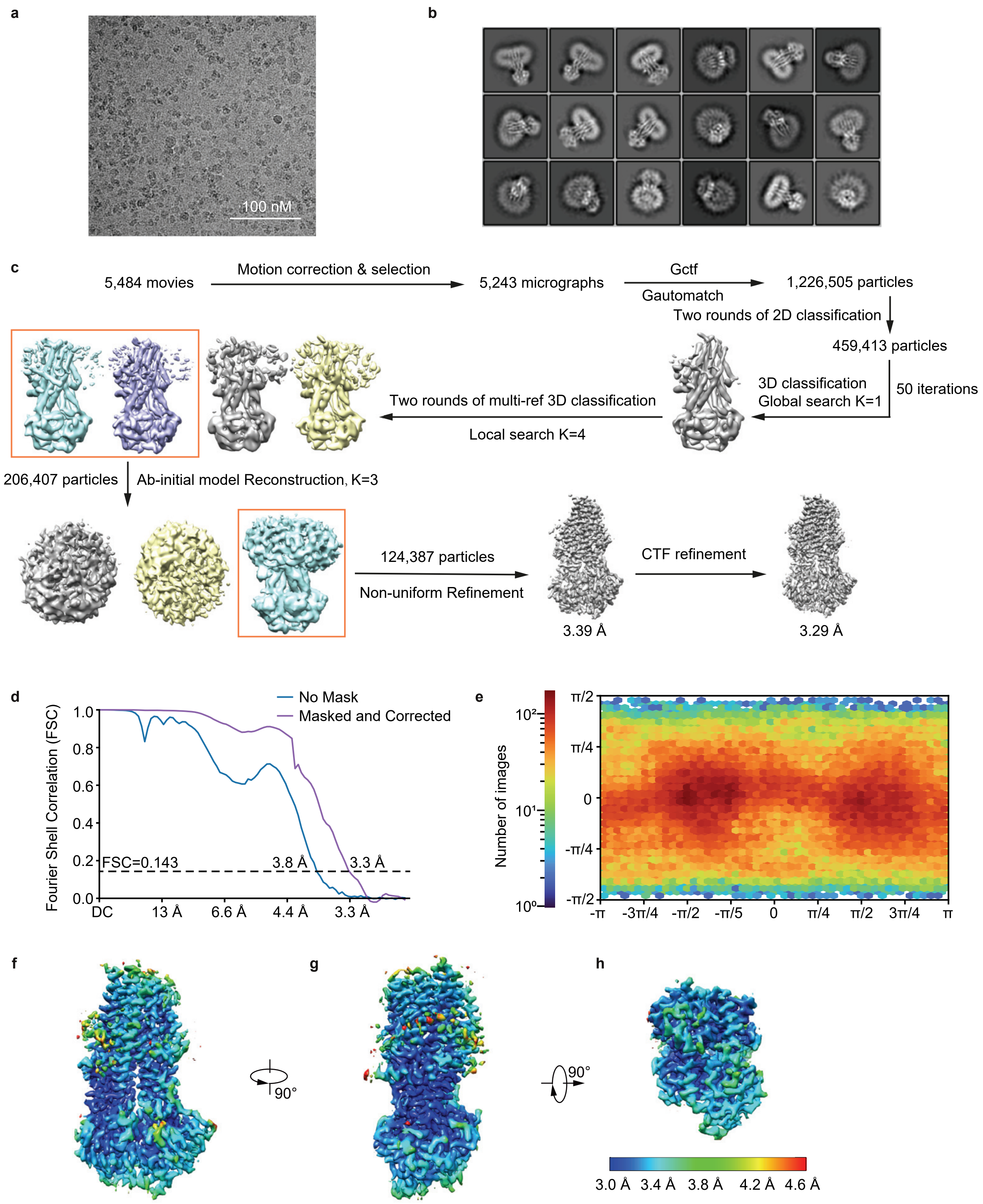

Supplementary Fig. 3: Workflow for cryo-EM data processing of the SUR2B in complex with Mg-nucleotides and P1075. a Representative image from a dataset consisting of 5,243 motion corrected micrographs. Experimet was repeated independently more than 3 times with similar results. **b** Representative two-dimensional class averages of SUR2B. **c** EM data processing workflow. **d** Resolution estimation of the SUR2B map, based on the criterion of the FSC 0.143 cut-off. **e** Angular distribution of the final reconstruction. **f-h** Local resolution map of the final density map.

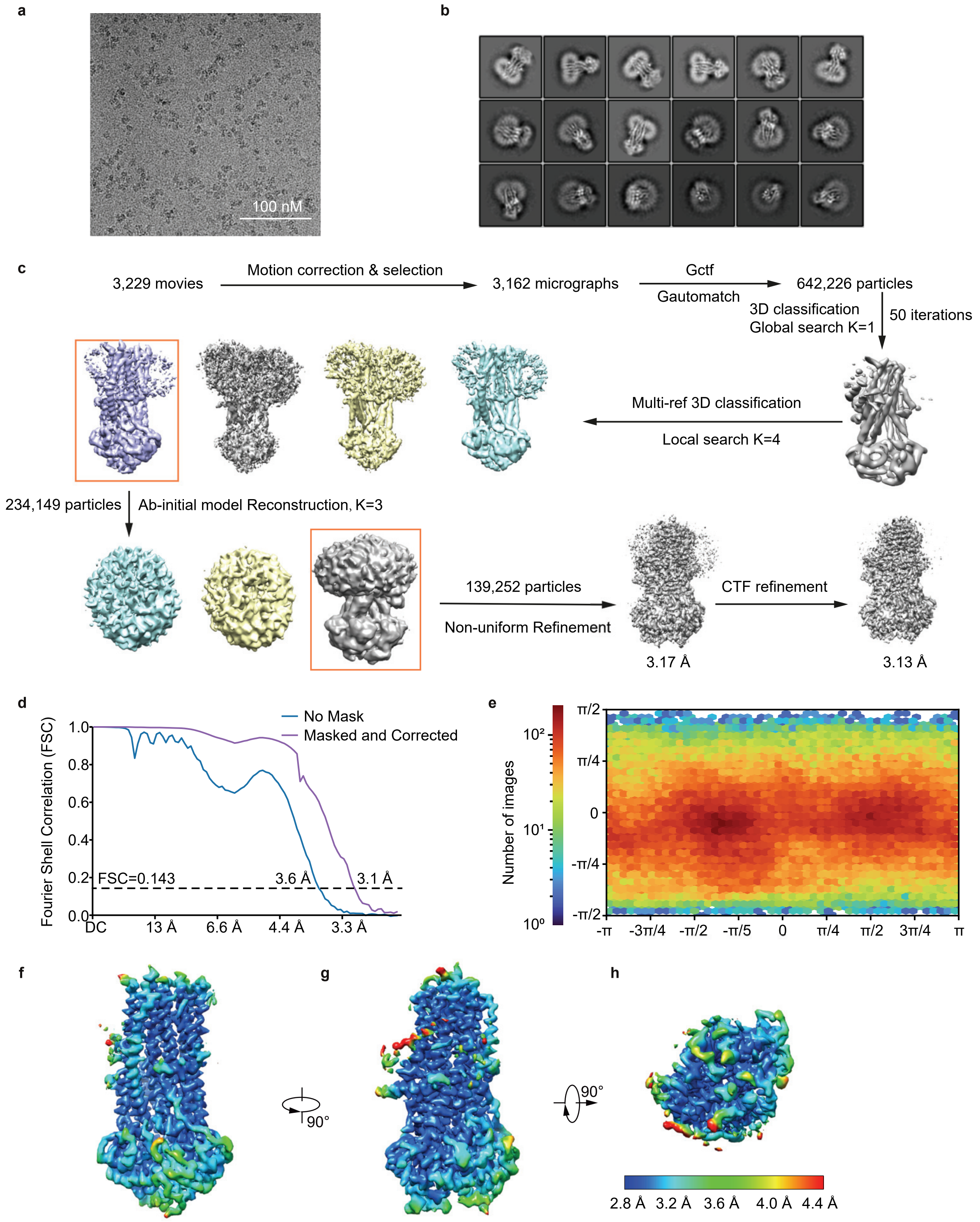

Supplementary Fig. 4: Workflow for cryo-EM data processing of the SUR2B in complex with Mg-nucleotides and Lev. a Representative image from a dataset consisting of 3,162 motion corrected micrographs. Experimet was repeated independently more than 3 times with similar results. **b** Representative two-dimensional class averages of SUR2B. **c** EM data processing workflow. **d** Resolution estimation of the SUR2B map, based on the criterion of the FSC 0.143 cut-off. **e** Angular distribution of the final reconstruction. **f-h** Local resolution map of the final density map.

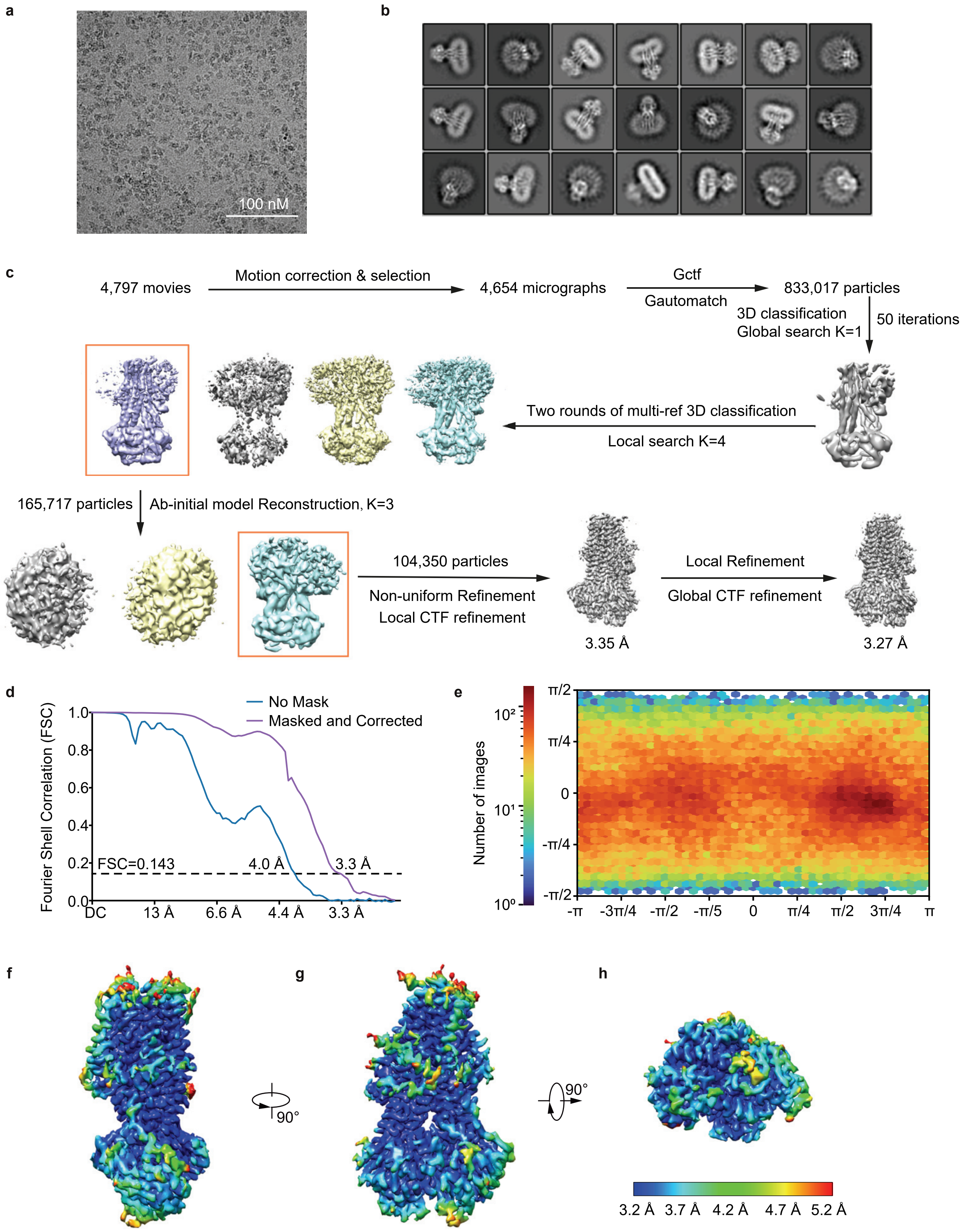

Supplementary Fig. 5: Workflow for cryo-EM data processing of the SUR2A in complex with Mg-nucleotides and P1075. a Representative image from a dataset consisting of 4,654 motion corrected micrographs. Experimet was repeated independently more than 3 times with similar results. **b** Representative two-dimensional class averages of SUR2A. **c** EM data processing workflow. **d** Resolution estimation of the SUR2A map, based on the criterion of the FSC 0.143 cut-off. **e** Angular distribution of the final reconstruction. **f-h** Local resolution map of the final density map.

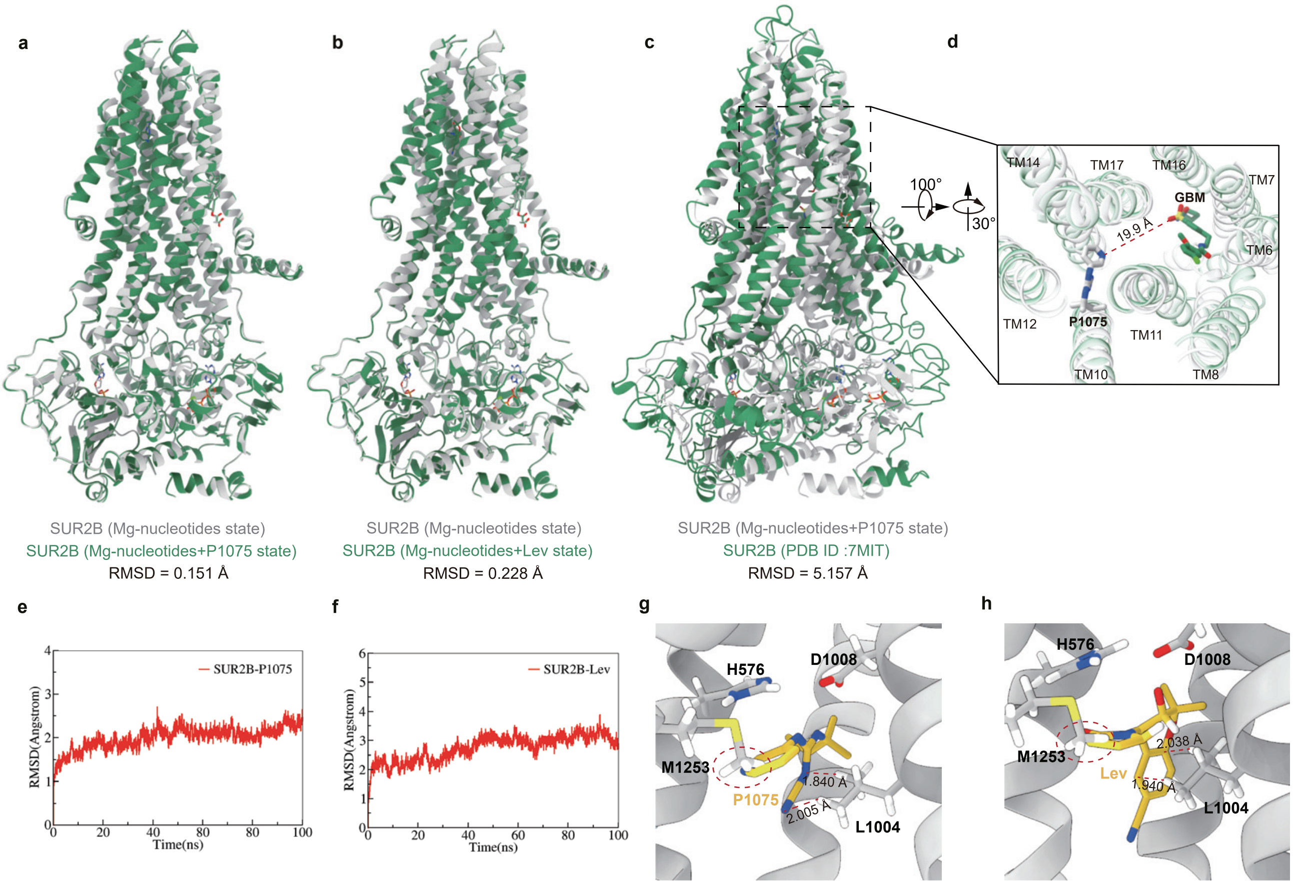

Supplementary Fig. 6: Structural comparison of SUR2B with different ligands. a Structural alignment of SUR2B in Mg-nucleotides state (gray) and Mg-nucleotides + P1075 state (green). **b** Structural alignment of SUR2B in Mg-nucleotides state (gray) and Mg-nucleotides + Lev state (green). **c** Structural alignment of SUR2B in Mg-nucleotides + P1075 state (gray) and inward-facing state (PDB ID: 7MIT, green). **d** Close-up view of P1075 and GBM binding sites boxed in **c**. Distance between N4 of P1075 and O4 of GBM is shown as dashed line. **e,f** MD simulation of P1075 **(e)** and Lev **(f)** binding to SUR2B. **g** Sterical clashes between M1253 mutant and P1075 or between L1004 mutant and P1075. Steric clashes of M1253 and P1075 are outlined by red dashed circles. **h** Sterical clashes between M1253 mutant and Lev or between L1004 mutant and Lev. Steric clashes of M1253 and Lev are outlined by red dashed circles.

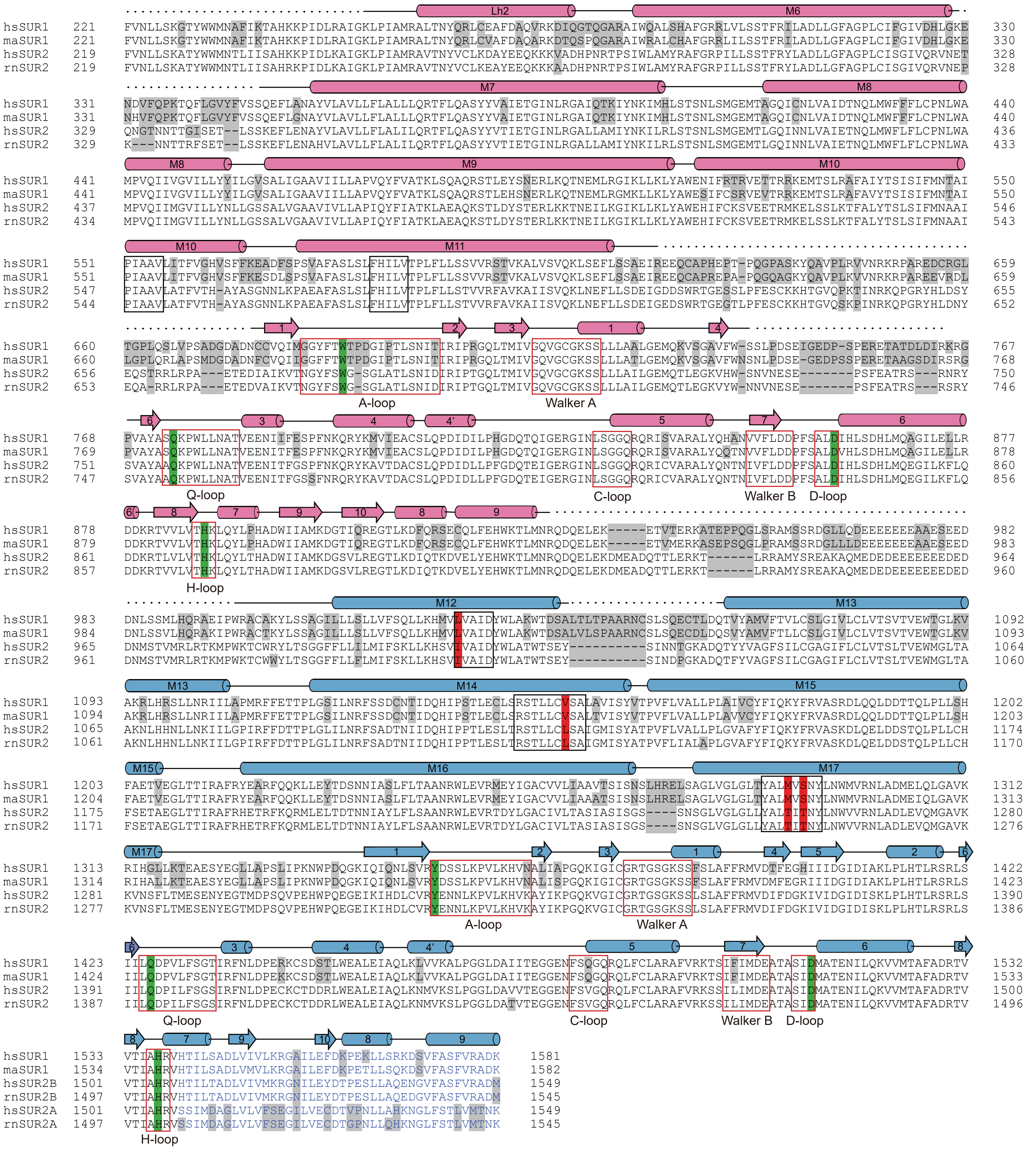

Supplementary Fig. 7: Sequence alignment of SUR from different species. Sequence alignment of *Homo sapiens* SUR1 (hsSUR1), *Mesocricetus auratus* SUR1 (maSUR1), *Homo sapiens* SUR2 (hsSUR2) and *Rattus norvegicus* SUR2 (rnSUR2). Secondary structure elements are shown as cylinders (α helices), arrows (β sheets), and lines (loops). Non-conserved residues are highlighted in gray. Residues of the KCOs binding sites are boxed by black lines and different residues between SUR1 and SUR2 are highlighted in red. Key motifs in NBDs are boxed by red lines and key residues are highlighted in green. The C-terminal 42 residues (C42) were colored in blue.

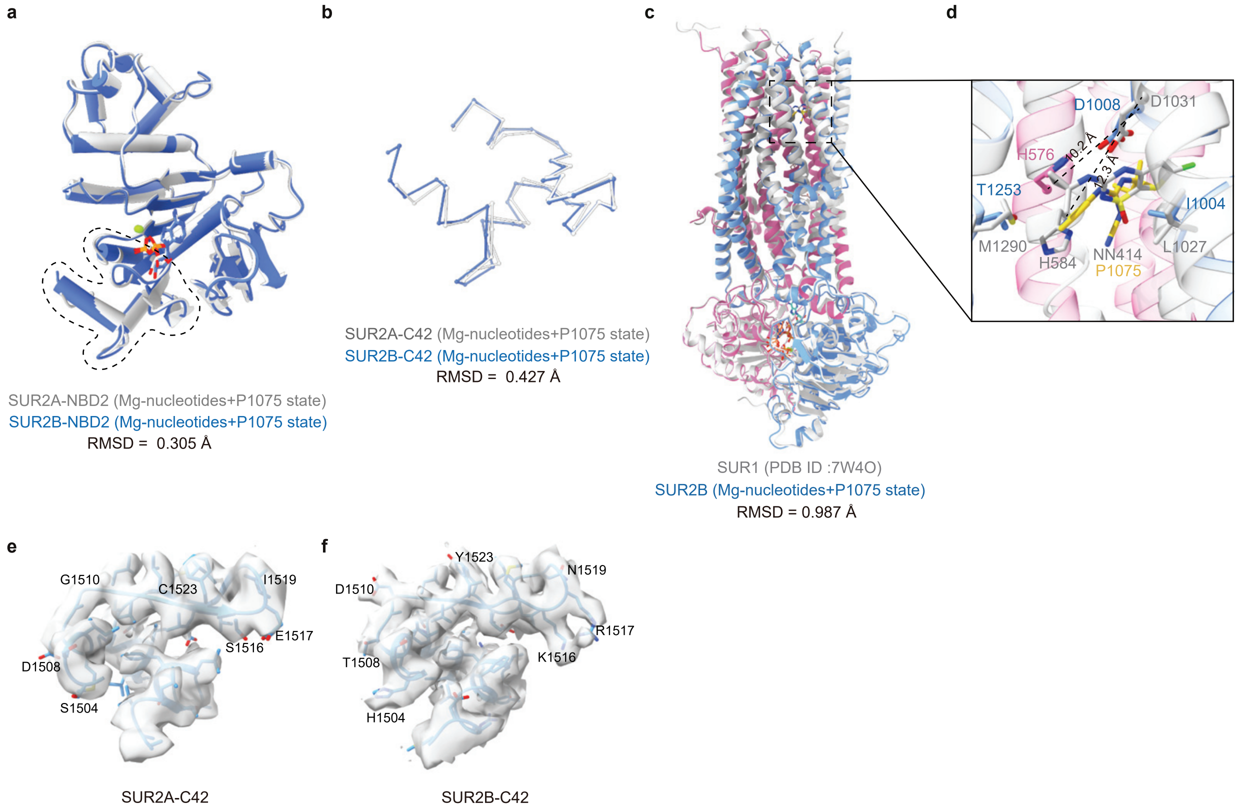

Supplementary Fig. 8: Structural comparison of SUR in different states. a Structural comparison of SUR2A-NBD2 (gray) and SUR2B-NBD2 (blue) in complex with Mg-nucleotides and P1075. **b** Close-up view of the structural comparison of the The C-terminal 42 residues (C42) between SUR2A and SUR2B in **a**. **c** Structure alignment of SUR1 in Mg-nucleotides + NN414 state (gray) and Mg-nucleotides + P1075 state (colored). **d** Close-up view of P1075 and NN414 binding sites in **c**. Cα distances between H576(H584 in SUR1) and D1008(D1031 in SUR1) are shown as dashed lines. **e-f** Cryo-EM density of C-terminal 42 residues of SUR2A (e) and SUR2B (f). Residues of SUR2 are shown as sticks.

Supplementary Table 1: Cryo-EM data collection, refinement and validation statistics

	SUR2B Mg-ADP/ATP	SUR2B Mg-ADP/ATP + P1075	SUR2B Mg-ADP/ATP + Lev	SUR2A Mg-ADP/ATP + P1075
PDB ID	7VLR	7VLS	7VLT	7VLU
EMDB ID	EMD-32024	EMD-32025	EMD-32026	EMD-32027
**Data collection and processing**				
Magnification	165,000 ×	165,000 ×	165,000 ×	165,000 ×
Voltage (kV)	300	300	300	300
Electron exposure ($e^-/Å^2$)	50	50	50	50
Defocus range (set) (μm)	-1.5 to -2.0	-1.5 to -2.0	-1.5 to -2.0	-1.5 to -2.0
Pixel size (Å)	0.821	0.821	0.821	0.821
Symmetry imposed	*C1*	*C1*	*C1*	*C1*
Initial particle images (no.)	389,430	1,226,505	642,226	833,017
Final particle images (no.)	36,180	124,387	139,252	104,350
Map resolution (Å)	3.4	3.3	3.1	3.3
FSC threshold	0.143	0.143	0.143	0.143
Map resolution range (Å)	250-3.4	250-3.3	250-3.1	250-3.3
**Refinement**				
Initial model used (PDB code)	5YWD	5YWD	5YWD	5YWD
Model resolution (Å)	3.4	3.3	3.1	3.3
FSC threshold	0.143	0.143	0.143	0.143
Model resolution range (Å)	250-3.3	250-3.2	250-3.1	250-3.2
Map sharpening B factor ($Å^2$)	-117.7	-142.6	-139.4	-120.6
Model composition				
Non-hydrogen atoms	9,003	9,003	9,024	9,110
Protein residues	1,145	1,141	1,145	1,159
Ligands	5	6	6	6
B factors ($Å^2$)				
Protein	52.53	52.61	58.36	45.95
Ligand	58.72	54.50	68.42	53.41
R.m.s. deviations				
Bond lengths (Å)	0.005	0.004	0.004	0.004
Bond angles (°)	0.986	0.988	0.998	0.993
Validation				
MolProbity score	1.16	1.15	1.31	1.38
Clashscore	3.70	1.6	2.65	2.67
Poor rotamers (%)	0.94	1.46	2.19	1.96
Ramachandran plot				
Favored (%)	98.41	97.43	98.06	97.47
Allowed (%)	1.59	2.57	1.94	2.53
Disallowed (%)	0.00	0.00	0.00	0.00
