# Supplementary material for: Structural identification of vasodilator binding sites on the SUR2 subunit
Source: Nat Commun. 2022 May 13;13:2675. doi: 10.1038/s41467-022-30428-y (PMC9106677; doi:10.1038/s41467-022-30428-y)
Supplement: Supplementary file 2 — Reporting Summary [file 41467_2022_30428_MOESM2_ESM.pdf]

nature portfolio

Corresponding author(s): Lei Chen

Last updated by author(s): 2022-04-05

Reporting Summary

Nature Portfolio wishes to improve the reproducibility of the work that we publish. This form provides structure for consistency and transparency in reporting. For further information on Nature Portfolio policies, see our Editorial Policies and the Editorial Policy Checklist.

Statistics

For all statistical analyses, confirm that the following items are present in the figure legend, table legend, main text, or Methods section.

n/a	Confirmed	
☐	☒	The exact sample size (n) for each experimental group/condition, given as a discrete number and unit of measurement
☐	☒	A statement on whether measurements were taken from distinct samples or whether the same sample was measured repeatedly
☒	☐	The statistical test(s) used AND whether they are one- or two-sided<br>*Only common tests should be described solely by name; describe more complex techniques in the Methods section.*
☒	☐	A description of all covariates tested
☒	☐	A description of any assumptions or corrections, such as tests of normality and adjustment for multiple comparisons
☐	☒	A full description of the statistical parameters including central tendency (e.g. means) or other basic estimates (e.g. regression coefficient) AND variation (e.g. standard deviation) or associated estimates of uncertainty (e.g. confidence intervals)
☒	☐	For null hypothesis testing, the test statistic (e.g. F, t, r) with confidence intervals, effect sizes, degrees of freedom and P value noted<br>*Give P values as exact values whenever suitable.*
☒	☐	For Bayesian analysis, information on the choice of priors and Markov chain Monte Carlo settings
☒	☐	For hierarchical and complex designs, identification of the appropriate level for tests and full reporting of outcomes
☒	☐	Estimates of effect sizes (e.g. Cohen's d, Pearson's r), indicating how they were calculated

Our web collection on statistics for biologists contains articles on many of the points above.

Software and code

Policy information about availability of computer code

Data collection	SerialEM-3.6.11
Data analysis	MotionCor2-1.3.2, GCTF-1.18, Gautomatch-0.56, RELION 3.0, cryoSPARC-3.1.0, PHENIX-1.18rc1-3777, Coot-0.9.2, UCSF Chimera-1.14, Pymol-1.7.0.5, Graphpad Prism 5.0, UCSF ChimeraX-0.91, AMBER20, LeDock (version Omega), RDKit(version 2021.03.4), SHAKE algorithm

For manuscripts utilizing custom algorithms or software that are central to the research but not yet described in published literature, software must be made available to editors and reviewers. We strongly encourage code deposition in a community repository (e.g. GitHub). See the Nature Portfolio guidelines for submitting code & software for further information.

Data

Policy information about availability of data

All manuscripts must include a data availability statement. This statement should provide the following information, where applicable:

- Accession codes, unique identifiers, or web links for publicly available datasets
- A description of any restrictions on data availability
- For clinical datasets or third party data, please ensure that the statement adheres to our policy

The data that support the findings of this study are available from the corresponding author upon reasonable request. The structure data generated in this study have been deposited in EMDB and PDB as follows: SUR2B in complex with Mg-ATP/ADP: PDB: 7VLR [https://doi.org/10.2210/pdb7VLR/pdb], EMDB-32024 [https://www.ebi.ac.uk/pdbe/entry/emdb/EMD-32024]; SUR2B in complex with Mg-ATP/ADP and P1075: PDB: 7VLS [https://doi.org/10.2210/pdb7VLS/pdb], EMDB-32025 [https://www.ebi.ac.uk/pdbe/entry/emdb/EMD-32025]; SUR2B in complex with Mg-ATP/ADP and Lev: PDB: 7VLT [https://doi.org/10.2210/pdb7VLT/pdb], EMDB-32026 [https://www.ebi.ac.uk/pdbe/entry/emdb/EMD-32026] ; SUR2A in complex with Mg-ATP/ADP and P1075: PDB: 7VLU [https://doi.org/10.2210/pdb7VLU/pdb], EMDB-32027 [https://www.ebi.ac.uk/pdbe/entry/emdb/EMD-32027]. PDB ID: 7W4O, 5W81, 6UY0, 7MIT, 5YWD and 6JB1 are already available on [illegible]

Protein Data Bank. Source data are provided with this paper.

Field-specific reporting

Please select the one below that is the best fit for your research. If you are not sure, read the appropriate sections before making your selection.

☒ Life sciences ☐ Behavioural & social sciences ☐ Ecological, evolutionary & environmental sciences

For a reference copy of the document with all sections, see nature.com/documents/nr-reporting-summary-flat.pdf

Life sciences study design

All studies must disclose on these points even when the disclosure is negative.

Sample size	We did not predetermine the sample size. The sample sizes were chosen to ensure the reproducibility of the experiments and were wildly used in the field. The sample size were adequate based on the distribution of data and clearly visible effects. The numbers of biological independent sample were repeated at least 3 times and indicated in figure legends.
Data exclusions	Cryo-EM micrographs with ice or ethane contamination, empty carbon, and poor CTF fit (> 5 Å) were excluded manually. Particles belonging to bad classes were discarded and the data processing flowchart were summarized in Supplementary Figures. These criteria were pre-established and the procedure is a common practise in cryo-EM image analysis.
Replication	All attempts at replication were successful according to the detailed protocol described in the methods section. The numbers of replication were described in figure legends.
Randomization	For cryo-EM 3D refinement, all particles were randomly split into two groups. No group allocation was needed for functional experiments in this study.
Blinding	The investigators were blinded to group allocation during cryo-EM half map generation. Blinding is not relevant for protein structure determination and functional assays because these results are not subjective.

Reporting for specific materials, systems and methods

We require information from authors about some types of materials, experimental systems and methods used in many studies. Here, indicate whether each material, system or method listed is relevant to your study. If you are not sure if a list item applies to your research, read the appropriate section before selecting a response.

Materials & experimental systems

n/a	Involved in the study	
☒	☐	Antibodies
☐	☒	Eukaryotic cell lines
☒	☐	Palaeontology and archaeology
☒	☐	Animals and other organisms
☒	☐	Human research participants
☒	☐	Clinical data
☒	☐	Dual use research of concern

Methods

n/a	Involved in the study	
☒	☐	ChIP-seq
☒	☐	Flow cytometry
☒	☐	MRI-based neuroimaging

Eukaryotic cell lines

Policy information about cell lines

Cell line source(s)	Sf9 and HEK293F cells were from Thermo Fisher Scientific. AD293 cell was from Agilent.
Authentication	None of the cell line used was authenticated.
Mycoplasma contamination	All cell lines were tested negative for mycoplasma contamination.
Commonly misidentified lines (See ICLAC register)	No commonly misidentified cell lines were used.
